# Circulating liver function markers and the risk of COPD in the UK Biobank

**DOI:** 10.3389/fendo.2023.1121900

**Published:** 2023-03-22

**Authors:** Wencong Du, Haoyu Guan, Xinglin Wan, Zheng Zhu, Hao Yu, Pengfei Luo, Lulu Chen, Jian Su, Yan Lu, Dong Hang, Ran Tao, Ming Wu, Jinyi Zhou, Xikang Fan

**Affiliations:** ^1^ Department of Non-Communicable Chronic Disease Control, Jiangsu Provincial Center for Disease Control and Prevention, Nanjing, Jiangsu, China; ^2^ Department of Epidemiology, School of Public Health, Nanjing Medical University, Nanjing, China; ^3^ Department of Chronic Disease Prevention and Control, Suzhou City Center for Disease Control and Prevention, Suzhou, China

**Keywords:** chronic obstructive pulmonary disease, liver function, serum biomarker, prospective cohort study, liver-lung axis

## Abstract

**Objective:**

To investigate the associations of circulating liver function marker levels with the risk of chronic obstructive pulmonary disease (COPD).

**Methods:**

We leveraged the data of 372,056 participants from the UK Biobank between 2006 and 2010. The assessed circulating liver function markers included alanine aminotransferase (ALT), aspartate aminotransferase (AST), gamma-glutamyl transferase (GGT), alkaline phosphatase (ALP), total bilirubin (TBIL), albumin (ALB), and total protein (TP). Incident COPD was identified through linkage to the National Health Service registries. Cox proportional hazards models were used to estimate hazard ratios (HRs) and 95% confidence intervals (CIs).

**Results:**

During a median follow-up period of 12.3 (interquartile range:11.4-13.2) years, we documented 10,001 newly diagnosed COPD cases. Lower levels of ALT, TBIL, ALB, and TP and higher levels of GGT and ALP were nonlinearly associated with elevated COPD risk. The HR (95% CI) for decile 10 vs. 1 was 0.92 (0.84-1.01) for ALT, 0.82 (0.75-0.89) for TBIL, 0.74 (0.67-0.81) for ALB, 0.96 (0.88-1.04) for TP, 1.45 (1.31-1.62) for GGT, and 1.31 (1.19-1.45) for ALP. Restricted cubic spline analyses suggested a U-shaped relationship between AST levels and COPD risk (*P* for nonlinearity <0.05).

**Conclusion:**

We observed that all seven circulating liver function markers were nonlinearly associated with the risk of COPD, indicating the importance of liver function in COPD.

## Introduction

Chronic obstructive pulmonary disease (COPD) is a severe chronic disease characterized by persistent respiratory symptoms and airflow limitation (1). The pathological changes observed in COPD include chronic inflammation, and structural changes resulting from repeated injury and repair (2). Recent evidence suggests that increased innate immune response contributes directly to the risk of COPD (3–5). It has been shown that the liver is essential in immune regulation and actively participates in the innate immune response of systemic organs through the secretion of inflammatory cytokines and mediators (6). Some prospective studies and animal models have shown associations of circulating liver function markers with COPD exacerbations and mortality (6–10). These findings support the hypothesis of the ‘liver-lung axis’ that may affect COPD progression (6). However, direct evidence of the relationship between liver function and COPD risk remains limited.

Circulating liver function markers include alanine aminotransferase (ALT), aspartate aminotransferase (AST), gamma-glutamyl transferase (GGT), alkaline phosphatase (ALP), total bilirubin (TBIL), albumin (ALB), and total protein (TP). These biomarkers provide indicators of liver injury, metabolism, immunity, and nutrition. In the UK primary care study, higher bilirubin levels were associated with a reduced risk of respiratory disease (8); a prospective Korean study with a 13-year follow-up suggested that low ALT levels may be an underlying factor in COPD development (9). Additionally, a case-control study reported significant changes in the levels of ALT, AST, GGT, and ALP among COPD patients compared with healthy nonsmokers (11). However, previous studies were conducted only on single or partial liver function markers and were mostly case-control studies. There is still a lack of systematic analysis on the associations between liver function markers and COPD risk in the general population.

Therefore, we leveraged the data from the UK Biobank, a large prospective cohort study, to systematically examine the associations between circulating liver function markers (ALT, AST, GGT, ALP, TBIL, ALB, and TP) and COPD risk.

## Methods

### Study population

The UK Biobank is a population-based cohort that includes over a half million individuals aged between 40 and 69 years from 22 assessment centres across England, Scotland, and Wales. All participants completed touchscreen questionnaires on sociodemographics, personal medical history, and lifestyle factors, and physical measurements were conducted by trained nurses between 2006 and 2010. Blood samples were collected from all individuals at the time of recruitment and from approximately 18,000 individuals at repeat assessments in 2012-2013. The current study was conducted under UK Biobank application number 84525.

In our study, we excluded 3,516 participants with prevalent COPD, 8,349 participants with emphysema or chronic bronchitis at baseline, 41,779 participants with forced expiratory volume at 1 second/forced vital capacity (FEV_1_/FVC) <lower limit of normal (LLN) according to the Global Lung Function Initiative (GLI) 2012 criteria (12), 68,164 participants lacking data for any of the seven circulating liver function markers, and 12,460 participants who were identified as outliers according to the Studentized Deviate Many-Outlier procedure (13). In total, 372,056 participants were included in this study (**Supplementary Figure 1**).

### Assessment of circulating liver function markers

Levels of circulating liver function markers, including ALT, AST, GGT, TBIL, ALP, TP, and ALB, were measured by biochemical assays utilizing the Beckman Coulter AU5800 Platform. ALT, AST, GGT, and ALP were analysed using the enzymatic method; TBIL, TP, and ALB were analyzed using the colorimetric method; high sensitivity C-reactive protein (CRP) was analysed using the immunoturbidimetric method. To reduce potential systematic error, the UK Biobank performed standardized procedures with strict quality assurance such that blood samples were collected, transported, processed, and stored in the same way. The protocol detailing the process and storage of blood samples has been extensively validated (14). Moreover, third-party internal quality control (IQC) materials were run for biochemical assays to provide an unbiased assessment of the analytical procedures. The average coefficients of variation (%) across high, medium, and low IQC levels of ALT, AST, GGT, ALP, TBIL, ALB, TP, and CRP were 1.2-2.9, 1.3-2.1, 1.4-2.8, 2.8-3.1,1.5-1.9, 2.1-2.2, 1.1-1.2, and 1.7-2.3, respectively. The details about circulating liver function marker assay performance have been described in the online UK Biobank Showcase (https://biobank.ndph.ox.ac.uk/showcase/ukb/docs/serum_biochemistry.pdf).

### Ascertainment of COPD

Incident COPD cases and deaths were identified through linkage to the National Health Service registries and national death registries. COPD was defined as J40-44 according to the 10th revision of the International Classification of Diseases (ICD-10).

### Statistical analysis

Person-years of follow-up were calculated from the baseline assessment to the first COPD diagnosis, death, loss to follow-up, or the end of the study (30 September 2021 for England, 31 July 2021 for Scotland, and 28 February 2018 for Wales), whichever occurred first. Intraclass correlation coefficients (ICCs) were calculated to evaluate the consistency of repeatedly measured circulating liver function marker levels. We used the Cox proportional hazards model to estimate the hazard ratios (HRs) and 95% confidence intervals (CIs), with age as the timescale. Model 1 was adjusted for sex, race, fasting status, assessment centre, and age at recruitment. Model 2 was further adjusted for education level, Townsend deprivation index, body mass index (BMI), physical activity, alcohol consumption frequency, smoking status, C-reactive protein, family history of respiratory disease, passive smoking, fine particulate matter (PM_2.5_), and occupations at risk of COPD. Model 3 further used ICCs to recalibrate the multivariate estimates.

Restricted cubic splines with 4 knots were used to fully assess the nonlinear associations between circulating liver function markers and COPD risk. The likelihood ratio test was used to compare the model with only the linear term of liver function marker levels to the model with both the linear term and cubic spline term. *P <*0.05 indicated the statistical significance of nonlinearity. Stratified analyses were conducted according to age at recruitment (<60, ≥60 years), sex (female, male), smoking status (never, previous, current), alcohol consumption (never or special occasions only, once a month to twice a week, three times a week to daily), BMI (<25, 25-30, ≥30 kg/m^2^), PM_2.5_ (<9.83, ≥9.83 μg/m^3^) (15), and occupations at risk of COPD (not at risk, at risk) (16). Multiplicative interactions were calculated using the likelihood ratio test to compare models with and without the cross-product terms.

In sensitivity analyses, we excluded participants who were diagnosed with COPD in the first two years of follow-up (n = 735), who had abnormally low or high levels of circulating liver function markers, who had hepatitis and other liver/hepatobiliary diseases (n = 8,680), who had asthma (n = 37,573), who had atherosclerotic diseases (n = 8,199), and who had diabetes at recruitment (n = 7,413). Moreover, we additionally adjusted cholesterol lowering medication including statins, etc. We also adjusted dietary and nutritional supplement intake, including frequency of poultry and livestock consumption, raw and cooked vegetable, fresh and dried fruit, coffee, and vitamin, mineral, and other dietary supplement intake.

All analyses were performed using SAS 9.4 (SAS Institute, Cary, NC). Statistical tests were all two-sided, and *P <*0.05 was defined as statistically significant.

## Results

During a median follow-up period of 12.3 years (interquartile range:11.4-13.2), we documented 10,001 incident COPD cases among 372,056 participants. Individuals with COPD were older and more likely to be current smokers, had higher levels of physical activity, and BMI and had a higher prevalence of asthma (**Table 1**). All seven circulating liver function markers were generally within the normal range, with the lowest percentage of participants within a normal range being 84.58% for ALP (**Supplementary Table 1**). In Spearman correlation analysis, ALT and AST (*rs* = 0.69) and ALT and GGT (*rs* = 0.58) were strongly correlated (**Supplementary Table 2**). The ICCs value between the two measurements of these biomarkers were 0.50 (95% CI: 0.49-0.51) for ALT, 0.49 (95% CI: 0.47-0.50) for AST, 0.61 (95% CI: 0.59-0.62) for GGT, 0.72 (95% CI: 0.71-0.73) for ALP, 0.73 (95% CI: 0.73-0.74) for TBIL, 0.46 (95% CI: 0.44-0.47) for ALB, and 0.46 (95% CI: 0.44-0.47) for TP (**Supplementary Table 3**).

**Table 1:** Characteristics of study participants at baseline.

Characteristics^a^	Total cohort	Participants without COPD	Participants with incident COPD	*P* values
N	372056	362055	10001	
Age at recruitment (years)	56.50 (8.08)	56.37 (8.08)	61.18 (6.53)	<0.0001
Male sex (%)	167759 (45.09)	162492 (44.88)	5267 (52.66)	<0.0001
White race (%)	351462 (94.46)	341849 (94.42)	9613 (96.12)	<0.0001
Fasting status (%)				<0.0001
<8 hours	357491 (96.09)	348072 (96.14)	9419 (94.18)	
≥8 hours	14565 (3.91)	13983 (3.86)	582 (5.82)	
College/university education (%)	122708 (32.98)	121170 (33.47)	1538 (15.38)	<0.0001
Townsend deprivation index	-1.41 (3.03)	-1.44 (3.01)	-0.17 (3.47)	<0.0001
Smoking status (%)^b^				<0.0001
Never	208496 (56.04)	206250 (56.97)	2246 (22.46)	
Previous	127520 (34.27)	122890 (33.94)	4630 (46.30)	
Current, pack-years <10	4614 (1.24)	4442 (1.23)	172 (1.72)	
Current, pack-years ≥10 and <20	6538 (1.76)	6148 (1.70)	390 (3.90)	
Current, pack-years ≥20 and <30	5983 (1.61)	5420 (1.50)	563 (5.63)	
Current, pack-years ≥30	9375 (2.52)	7754 (2.14)	1621 (16.21)	
Alcohol consumption frequency (%)^b^				<0.0001
Never or special occasions only	71102 (19.11)	68279 (18.86)	2823 (28.23)	
Once a month to twice a week	139173 (37.41)	135747 (37.49)	3426 (34.26)	
Three times a week to daily	161024 (43.28)	157315 (43.45)	3709 (37.09)	
Body mass index (kg/m^2^)	27.45 (4.75)	27.40 (4.71)	28.99 (5.63)	<0.0001
Physical activity (MET hours per week)	15.61 (20.41)	15.57 (20.36)	16.91 (22.12)	<0.0001
Prevalent asthma cases (%)	37573 (10.10)	34927 (9.65)	2646 (26.46)	<0.0001
Prevalent diabetes cases (%)	7413 (1.99)	6778 (1.87)	635 (6.35)	<0.0001
Prevalent liver disease cases (%)	8680 (2.33)	8208 (2.27)	472 (4.72)	<0.0001
Prevalent atherosclerotic disease cases (%)	8199 (2.20)	7450 (2.06)	749 (7.49)	<0.0001
Family history of respiratory disease (%)	55073 (14.80)	52505 (14.50)	2568 (25.68)	<0.0001
Passive smoking (%)^b^				<0.0001
Never	248296 (66.74)	243812 (67.34)	4484 (44.84)	
<20 hours a week	66129 (17.77)	64309 (17.76)	1820 (18.20)	
≥20 hours a week	4031 (1.08)	3850 (1.06)	181 (1.81)	
PM_2.5_ (μg/m^3^)	9.97 (1.05)	9.96 (1.05)	10.19 (1.10)	<0.0001
Occupations at risk of COPD (%)	7001 (1.88)	6750 (1.86)	251 (2.51)	<0.0001
Alanine aminotransferase (ALT; U/L)	22.54 (10.65)	22.54 (10.65)	22.80 (10.62)	0.01
Aspartate aminotransferase (AST; U/L)	25.40 (6.64)	25.39 (6.62)	25.77 (7.18)	<0.0001
Gamma-glutamyl transferase (GGT; U/L)	33.10 (22.56)	32.92 (22.44)	39.49 (25.56)	<0.0001
Total bilirubin (TBIL; µmol/L)	8.97 (3.86)	8.98 (3.87)	8.43 (3.55)	<0.0001
Alkaline phosphatase (ALP; U/L)	82.41 (22.31)	82.21 (22.24)	89.84 (23.58)	<0.0001
Total protein (TP; g/L)	72.49 (4.05)	72.49 (4.05)	72.17 (4.27)	<0.0001
Albumin (ALB; g/L)	45.23 (2.59)	45.25 (2.59)	44.48 (2.69)	<0.0001

COPD, chronic obstructive pulmonary disease; MET, metabolic equivalents; PM_2.5_, fine particulate matter.

a Mean (SD) values and percentages are reported for continuous and categorical variables, respectively.

b The percentage of the categorical variables does not sum to 100% because some participants chose to “not answer”.

**Figure 1:**
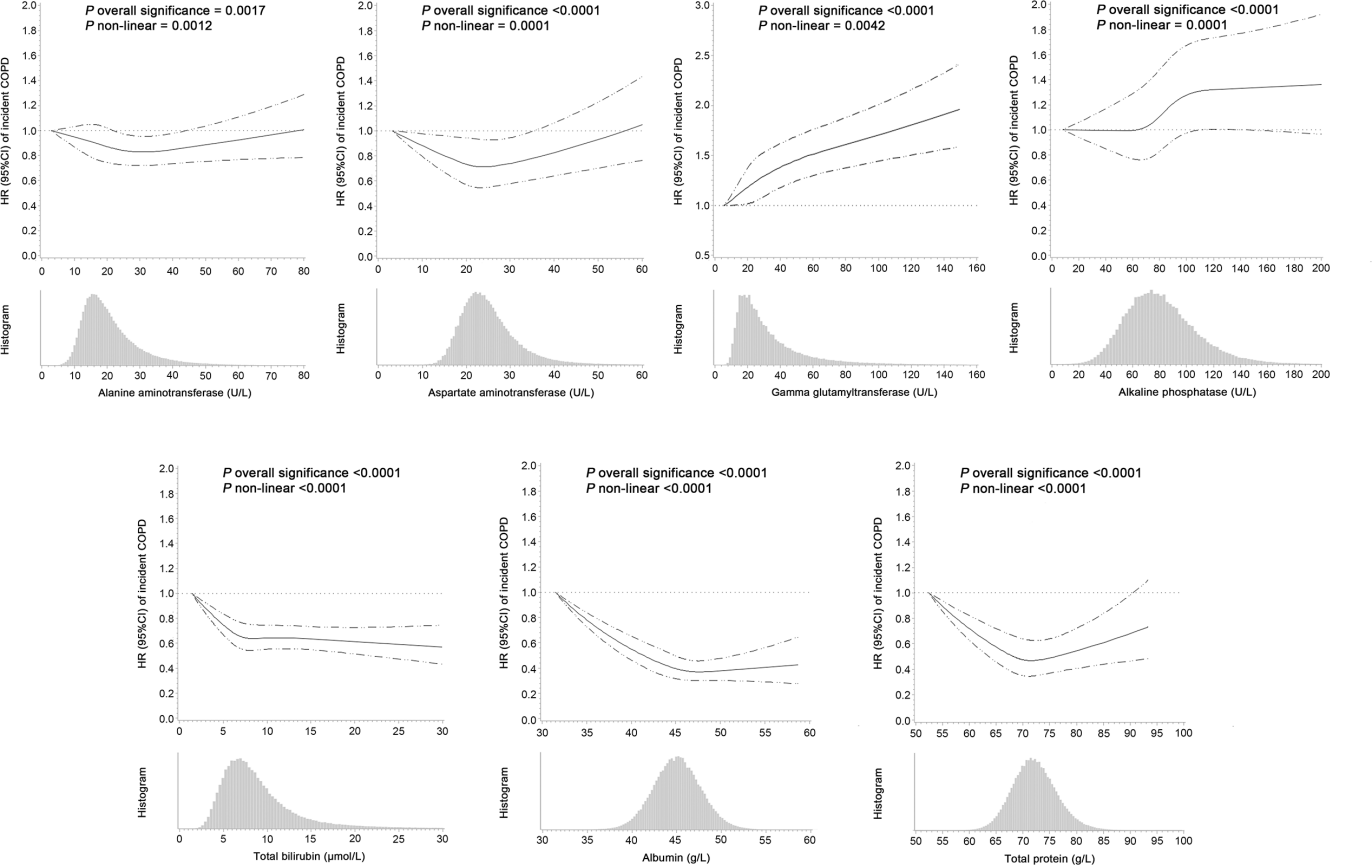
Hazard ratios (95% confidence intervals) of incident COPD associated with circulating levels of liver function markers and the frequency histograms of liver function markers in the UK Biobank. The associations were examined by multivariate Cox regression models based on restricted cubic splines. The solid line represents estimates of hazard ratios and dashed lines represent 95% confidence intervals.

In the multivariate adjusted model, we found that lower levels of ALT, TBIL, ALB, TP, and higher levels of GGT and ALP were associated with an elevated risk of COPD. AST levels showed a U-shaped association with COPD risk (**Table 2** and **Figure 1**). The corresponding HRs (95% CIs) for deciles 10 vs. 1 were 0.92 (0.84-1.01) for ALT, 0.82 (0.75-0.89) for TBIL, 0.74 (0.67-0.81) for ALB, 0.96 (0.88-1.04) for TP, 1.45 (1.31-1.62) for GGT, and 1.31 (1.19-1.45) for ALP. After correction for ICCs, the associations between circulating liver function markers and COPD risk became stronger (**Table 2**). Restricted cubic spline analyses showed a nonlinear relationship between these biomarkers and COPD risk (all *P* for nonlinearity <0.05) (**Figure 1**).

**Table 2:** HRs (95% CIs) of COPD associated with circulating levels of liver function markers.

	Decile 1	Decile2		Decile 3		Decile4			Decile 5			Decile 6		Decile 7		Decile 8		Decile 9		Decile 10		*P* for overall			*P* for nonlinearity	
Alanine aminotransferase (ALT)																										
Range	<12.3	12.3-14.4		14.4-16.2		16.2-18.0			18.0-19.9			19.9-22.2		22.2-25.0		25.0-29.0		29.0-36.1		≥36.1						
COPD cases	904	869		1001		1041			998			1068		1051		1043		1028		998						
HR (95% CI)^a^	1 (reference)	0.86 (0.78-0.94)		0.91 (0.83-1.00)		0.89 (0.81-0.97)			0.83 (0.76-0.91)			0.88 (0.80-0.96)		0.85 (0.78-0.93)		0.86 (0.78-0.94)		0.87 (0.79-0.95)		0.94 (0.86-1.03)		<0.0001			<0.0001	
HR (95% CI)^b^	1 (reference)	0.92 (0.84-1.02)		0.99 (0.90-1.08)		0.97 (0.88-1.06)			0.89 (0.81-0.97)			0.94 (0.86-1.03)		0.90 (0.82-0.98)		0.89 (0.81-0.97)		0.87 (0.79-0.95)		0.92 (0.84-1.01)		0.001			0.002	
HR (95% CI)^c^	1 (reference)	0.85 (0.71-1.03)		0.98 (0.82-1.17)		0.94 (0.78-1.12)			0.78 (0.65-0.94)			0.89 (0.74-1.06)		0.81 (0.67-0.97)		0.79 (0.66-0.94)		0.75 (0.63-0.91)		0.85 (0.70-1.02)						
Aspartate aminotransferase (AST)																										
Range	<18.3	18.3-20.1		20.1-21.6		21.6-22.9			22.9-24.2			24.2-25.6		25.6-27.4		27.4-29.7		29.7-33.6		≥33.6						
COPD cases	1006	917		1030		972			952			927		1038		968		1034		1157						
HR (95% CI)^a^	1 (reference)	0.77 (0.70-0.84)		0.76 (0.70-0.83)		0.72 (0.66-0.78)			0.67 (0.61-0.73)			0.66 (0.60-0.72)		0.66 (0.61-0.72)		0.64 (0.59-0.70)		0.69 (0.63-0.75)		0.78 (0.72-0.85)		<0.0001			<0.0001	
HR (95% CI)^b^	1 (reference)	0.90 (0.82-0.99)		0.95 (0.87-1.04)		0.92 (0.84-1.01)			0.90 (0.82-0.98)			0.91 (0.83-0.99)		0.93 (0.85-1.01)		0.91 (0.83-0.99)		0.98 (0.90-1.08)		1.04 (0.95-1.13)		0.0001			0.0001	
HR (95% CI)^c^	1 (reference)	0.81 (0.67-0.97)		0.90 (0.75-1.07)		0.85 (0.71-1.01)			0.81 (0.67-0.97)			0.83 (0.69-0.99)		0.85 (0.71-1.02)		0.83 (0.69-0.99)		0.97 (0.81-1.16)		1.08 (0.90-1.29)						
Gamma-glutamyl transferase (GGT)																										
Range	<14.4	14.4-17.1		17.1-19.7		19.7-22.5			22.5-25.9			25.9-30.0		30.0-35.7		35.7-44.2		44.2-61.0		≥61.0						
COPD cases	489	603		751		813			1005			1040		1147		1235		1338		1580						
HR (95% CI)^a^	1 (reference)	1.05 (0.93-1.18)		1.22 (1.09-1.37)		1.27 (1.14-1.42)			1.48 (1.33-1.65)			1.57 (1.41-1.75)		1.68 (1.51-1.87)		1.85 (1.66-2.06)		1.99 (1.79-2.21)		2.44 (2.20-2.70)		<0.0001			<0.0001	
HR (95% CI)^b^	1 (reference)	0.96 (0.86-1.09)		1.03 (0.92-1.15)		1.03 (0.92-1.15)			1.10 (0.98-1.22)			1.15 (1.03-1.29)		1.15 (1.03-1.28)		1.22 (1.09-1.36)		1.24 (1.11-1.38)		1.45 (1.31-1.62)		<0.0001			0.004	
HR (95% CI)^c^	1 (reference)	0.94 (0.77-1.14)		1.04 (0.87-1.26)		1.04 (0.87-1.26)			1.16 (0.97-1.39)			1.26 (1.05-1.51)		1.25 (1.05-1.49)		1.38 (1.16-1.65)		1.42 (1.19-1.69)		1.85 (1.55-2.20)						
Alkaline phosphatase (ALP)																										
Range	<56.6	56.6-63.9		63.9-69.5		69.5-74.7			74.7-79.8			79.8-85.1		85.2-91.3		91.3-99.1		99.1-111.2		≥111.2							
COPD cases	529	631		738		833			875			1009		1122		1204		1420		1640							
HR (95% CI)^a^	1 (reference)	1.03 (0.92-1.15)		1.15 (1.03-1.28)		1.23 (1.10-1.37)			1.27 (1.14-1.42)			1.47 (1.32-1.63)		1.59 (1.44-1.77)		1.71 (1.54-1.89)		2.02 (1.82-2.23)		2.33 (2.11-2.57)		<0.0001			<0.0001		
HR (95% CI)^b^	1 (reference)	0.99 (0.88-1.11)		1.03 (0.92-1.15)		1.07 (0.96-1.19)			1.03 (0.92-1.15)			1.15 (1.03-1.28)		1.20 (1.08-1.33)		1.22 (1.10-1.35)		1.32 (1.19-1.46)		1.31 (1.19-1.45)		0.0001			<0.0001		
HR (95% CI)^c^	1 (reference)	0.98 (0.84-1.15)		1.04 (0.89-1.21)		1.09 (0.94-1.27)			1.04 (0.9-1.21)		1.21 (1.05-1.41)			1.29 (1.11-1.49)		1.31 (1.14-1.51)		1.47 (1.28-1.69)		1.46 (1.27-1.68)							
Total bilirubin (TBIL)																										
Range	<5.3	5.3-6.1		6.1-6.7		6.7-7.4			7.4-8.0		8.0-8.8			8.8-9.8		9.8-11.1		11.1-13.7		≥13.7							
COPD cases	1408	1141		1086		987			1003		946			906		836		897		791							
HR (95% CI)^a^	1 (reference)	0.72 (0.67-0.78)		0.66 (0.61-0.71)		0.59 (0.54-0.64)			0.57 (0.53-0.62)		0.52 (0.48-0.56)			0.49 (0.45-0.53)		0.44 (0.40-0.48)		0.46 (0.42-0.50)		0.41 (0.38-0.45)		<0.0001			<0.0001		
HR (95% CI)^b^	1 (reference)	0.86 (0.80-0.93)		0.87 (0.80-0.94)		0.84 (0.78-0.91)			0.86 (0.79-0.93)		0.82 (0.76-0.90)			0.82 (0.75-0.90)		0.77 (0.71-0.84)		0.86 (0.79-0.94)		0.82 (0.75-0.89)		0.0001			<0.0001		
HR (95% CI)^c^	1 (reference)	0.81 (0.73-0.91)		0.82 (0.74-0.92)		0.79 (0.71-0.88)			0.81 (0.72-0.91)		0.77 (0.68-0.86)			0.76 (0.68-0.86)		0.70 (0.62-0.79)		0.81 (0.72-0.91)		0.76 (0.67-0.86)							
Albumin (ALB)																										
Range	<42.0	42.0-43.1		43.1-43.9		43.9-44.6			44.6-45.2		45.2-45.8			45.8-46.5		46.5-47.4		47.4-48.5		≥48.5							
COPD cases	1665	1295		1221		1000			1011		855			779		777		734		664							
HR (95% CI)^a^	1 (reference)	0.78 (0.73-0.84)		0.74 (0.69-0.80)		0.63 (0.58-0.68)			0.64 (0.59-0.69)		0.56 (0.51-0.61)			0.51 (0.47-0.56)		0.52 (0.48-0.57)		0.51 (0.47-0.56)		0.50 (0.46-0.55)		<0.0001			<0.0001		
HR (95% CI)^b^	1 (reference)	0.89 (0.83-0.96)		0.89 (0.82-0.95)		0.76 (0.71-0.83)			0.82 (0.75-0.88)		0.75 (0.69-0.81)			0.70 (0.64-0.76)		0.73 (0.67-0.80)		0.73 (0.67-0.80)		0.74 (0.67-0.81)		<0.0001			<0.0001		
HR (95% CI)^c^	1 (reference)	0.78 (0.66-0.91)		0.77 (0.65-0.90)		0.56 (0.47-0.66)			0.64 (0.54-0.76)		0.53 (0.44-0.64)			0.46 (0.38-0.55)		0.50 (0.42-0.61)		0.51 (0.42-0.62)		0.52 (0.42-0.63)							
Total protein (TP)																										
Range	<67.5	67.5-69.1		69.1-70.3		70.3-71.3			71.3-72.3		72.3-73.3			73.3-74.4		74.4-75.7		75.7-77.7		≥77.7							
COPD cases	1285	1064		1020		988			994		898			939		905		922		986							
HR (95% CI)^a^	1 (reference)	0.85 (0.78-0.92)		0.83 (0.76-0.90)		0.81 (0.75-0.88)			0.82 (0.76-0.89)		0.76 (0.69-0.82)			0.80 (0.73-0.87)		0.78 (0.71-0.85)		0.80 (0.74-0.87)		0.88 (0.81-0.95)		<0.0001			<0.0001	
HR (95% CI)^b^	1 (reference)	0.87 (0.80-0.94)		0.89 (0.82-0.96)		0.85 (0.78-0.93)			0.91 (0.84-0.99)		0.83 (0.76-0.90)			0.88 (0.81-0.96)		0.85 (0.78-0.93)		0.91 (0.83-0.99)		0.96 (0.88-1.04)		0.0001			<0.0001	
HR (95% CI)^c^	1 (reference)	0.73 (0.61-0.87)		0.77 (0.65-0.92)		0.70 (0.59-0.84)			0.81 (0.68-0.97)		0.66 (0.55-0.79)			0.76 (0.63-0.91)		0.71 (0.59-0.85)		0.81 (0.67-0.97)		0.91 (0.76-1.09)						

COPD, chronic obstructive pulmonary disease; HR, hazard ratio; CI, confidence interval.

a Model 1: Estimated from the Cox regression model with age as the underlying time scale and adjusted for sex, race (white, nonwhite, unknown), fasting status (<8 hours, ≥8 hours), assessment centres and age at recruitment (continuous).

b Model 2: Further adjusted for Townsend deprivation index (continuous), education level (college/university degree, noncollege/university degree, unknown), body mass index (continuous), total physical activity (continuous), alcohol consumption frequency (never or special occasions only, once a month to twice a week, three times a week to daily), smoking status (never, previous, current-pack-years <10, current-pack-years ≥10 and <20, current-pack-years ≥20 and <30, current-pack-years ≥30), C-reaction protein (continuous), family history of respiratory disease (no, yes, unknown), passive smoking (never, <20 hours a week, ≥20 hours a week), PM_2.5_ (continuous), occupations at risk of COPD (no, yes, unknown).

c Recalibrated multivariable estimates accounting for dilution bias using the intraclass correlation coefficients calculated in the subsample of participants with repeat measurements of circulating liver function markers.

Stratified analyses showed that the associations of ALP and ALB with COPD risk were stronger in male and younger participants. Interactions of ALT, AST, and GGT with smoking status were observed for COPD risk. Moreover, we found significant interactions of ALP, ALB, and TP with alcohol consumption and ALP, TBIL, and TP with BMI for the risk of COPD. The significant interactions of ALP with PM_2.5_ and occupations were also observed for COPD risk (all *P* for interaction <0.05) (**Table 3**).

**Table 3:** Stratified analyses of the association between circulating levels of liver function markers and COPD risk^a^.

	No. of COPD cases	ALT	AST	GGT	ALP	TBIL	ALB	TP
Age at recruitment								
< 60	3102	0.90 (0.77-1.05)	1.00 (0.87-1.16)	1.68 (1.40-2.02)	1.36 (1.14-1.62)	0.88 (0.75-1.03)	0.67 (0.57-0.78)	0.92 (0.79-1.07)
≥ 60	6899	0.94 (0.84-1.06)	1.07 (0.96-1.20)	1.34 (1.17-1.53)	1.29 (1.14-1.46)	0.81 (0.72-0.90)	0.77 (0.69-0.87)	0.98 (0.88-1.08)
***P* for interaction****^b^**		0.57	0.72	0.99	0.04	0.82	0.01	0.04
Sex								
Female	4734	0.98 (0.85-1.13)	1.00 (0.88-1.14)	1.54 (1.35-1.76)	1.18 (1.01-1.37)	0.81 (0.69-0.94)	0.79 (0.69-0.91)	0.96 (0.85-1.09)
Male	5267	0.93 (0.81-1.08)	1.08 (0.95-1.23)	1.23 (0.98-1.54)	1.52 (1.32-1.74)	0.81 (0.71-0.93)	0.69 (0.61-0.78)	0.97 (0.86-1.09)
***P* for interaction****^b^**		0.41	0.37	0.51	<0.0001	0.14	0.01	0.04
Smoking status								
Never	2246	0.77 (0.62-0.93)	0.82 (0.68-0.99)	1.36 (1.11-1.68)	1.21 (0.99-1.48)	0.86 (0.71-1.04)	0.74 (0.61-0.91)	1.04 (0.87-1.25)
Previous	4630	1.13 (0.97-1.31)	1.19 (1.04-1.37)	1.55 (1.32-1.83)	1.37 (1.18-1.58)	0.75 (0.65-0.86)	0.76 (0.66-0.86)	0.92 (0.81-1.04)
Current	3031	0.89 (0.73-1.03)	0.95 (0.81-1.11)	1.31 (1.07-1.61)	1.38 (1.13-1.69)	1.01 (0.84-1.21)	0.73 (0.62-0.86)	0.95 (0.81-1.11)
***P* for interaction****^b^**		<0.0001	<0.0001	<0.0001	0.06	0.39	0.93	0.61
Alcohol consumption								
Never or special occasions only	2823	0.89 (0.75-1.06)	0.99 (0.84-1.15)	1.47 (1.22-1.77)	1.18 (0.97-1.43)	0.75 (0.63-0.90)	0.94 (0.79-1.11)	0.94 (0.81-1.10)
Once a month to twice a week	3426	0.98 (0.83-1.15)	1.10 (0.94-1.27)	1.49 (1.24-1.78)	1.40 (1.16-1.68)	0.92 (0.79-1.07)	0.66 (0.56-0.78)	0.83 (0.72-0.96)
Three times a week to daily	3709	0.88 (0.75-1.04)	1.02 (0.88-1.19)	1.33 (1.09-1.62)	1.34 (1.15-1.57)	0.79 (0.67-0.92)	0.69 (0.59-0.80)	1.09 (0.95-1.25)
***P* for interaction****^b^**		0.31	0.43	0.69	0.01	0.05	0.01	0.0003
Body mass index (kg/m^2^)								
< 25	2331	1.08 (0.84-1.38)	1.15 (0.95-1.39)	1.74 (1.42-2.12)	1.41 (1.17-1.72)	0.86 (0.72-1.04)	0.79 (0.66-0.93)	1.16 (0.98-1.38)
25-30	4038	0.91 (0.78-1.06)	0.94 (0.82-1.08)	1.43 (1.21-1.70)	1.48 (1.26-1.74)	0.77 (0.66-0.89)	0.72 (0.62-0.83)	0.96 (0.84-1.09)
≥ 30	3632	0.97 (0.82-1.14)	1.04 (0.90-1.20)	1.12 (0.90-1.39)	1.10 (0.93-1.31)	0.86 (0.74-1.00)	0.71 (0.60-0.85)	0.83 (0.72-0.96)
***P* for interaction****^b^**		0.20	0.22	0.08	0.0002	0.01	0.42	0.005
PM_2.5_ (μg/m^3^)								
< Median^c^	4119	0.93 (0.80-1.07)	1.06 (0.92-1.22)	1.38 (1.17-1.63)	0.83 (0.72-0.96)	1.49 (1.27-1.74)	1.01 (0.88-1.15)	0.66 (0.57-0.76)
≥ Median^c^	5882	0.92 (0.81-1.04)	1.03 (0.92-1.14)	1.50 (1.31-1.73)	0.81 (0.72-0.91)	1.20 (1.06-1.37)	0.92 (0.83-1.03)	0.80 (0.71-0.90)
***P* for interaction****^b^**		0.08	0.86	0.58	0.85	<0.01	0.32	0.11
Occupations at risk of COPD								
Not at risk	4083	0.96 (0.83-1.12)	1.02 (0.89-1.17)	1.41 (1.19-1.66)	0.88 (0.76-1.01)	1.44 (1.23-1.68)	0.99 (0.87-1.13)	0.77 (0.67-0.89)
At risk	251	0.74 (0.39-1.38)	0.59 (0.35-1.02)	1.34 (0.72-2.49)	0.50 (0.23-1.11)	1.11 (0.59-2.10)	0.50 (0.29-0.86)	0.50 (0.26-0.97)
***P* for interaction****^b^**		0.8	0.27	0.06	0.9	<0.01	0.48	0.24

ALT, alanine aminotransferase; AST, aspartate aminotransferase; GGT, gamma-glutamyl transferase; ALP, alkaline phosphatase; TBIL, total bilirubin; ALB, albumin; TP, total Protein; BMI, body mass index; CRP, C-reactive protein.

a Hazard ratios (95% confidence intervals) of COPD decile 10 vs. 1 in circulating liver function markers in multivariable Cox regression model with age as the underlying time scale was used and adjusted for sex, race (white, nonwhite, unknown), fasting status (<8 hours, ≥8 hours), assessment centre and age at recruitment (continuous), Townsend deprivation index (continuous), education level (college/university degree, noncollege/university degree, unknown), body mass index (continuous), total physical activity (continuous), alcohol consumption frequency (never or special occasions only, once a month to twice a week, three times a week to daily), smoking status (never, previous, current-pack-years <10, current-pack-years ≥10 and <20, current-pack-years ≥20 and <30, current-pack-years ≥30), C-reaction protein (continuous), family history of respiratory disease (no, yes, unknown), passive smoking (never, <20 hours a week, ≥20 hours a week), PM_2.5_ (continuous), occupations at risk of COPD (no, yes, unknown).

b P for interaction was calculated using the likelihood ratio test for the product terms between these stratified variables and circulating levels of liver function markers.

c Median: 9.83μg/m^3^

In sensitivity analyses, the aforementioned associations were not materially altered after excluding incident COPD cases in the first two years of follow-up, participants with diabetes, participants with asthma, participants with atherosclerotic diseases, participants with liver/hepatobiliary disease at recruitment, or participants with abnormally low or high levels of liver function markers. Additionally, we adjusted for cholesterol lowering medication, dietary and nutritional supplement intake, and the associations remained robust (**Supplementary Table 4**).

## Discussion

The results from this large cohort of approximately half a million individuals indicated that ALT, TBIL, ALB, and TP levels were inversely associated with COPD risk. In contrast, positive associations were found for GGT and ALP with the risk of COPD. A U-shaped association is apparent between AST and COPD risk. After several comprehensive sensitivity analyses, the observed associations did not essentially change. Additionally, we observed effect modification by several stratification factors in the associations of circulating liver function markers with COPD risk. To our knowledge, none of the previous studies systematically evaluated the relationship between circulating liver function markers and COPD risk.

Our analysis suggested that the levels of ALT were inversely associated with COPD risk. Previous studies also reported that low ALT levels play an important role in COPD development and exacerbations (9, 17). In clinical practice, circulating ALT levels commonly represent a specific liver dysfunction and injury marker. Recent studies indicated that low ALT activity has been linked to sarcopenia, frailty, and overall health (18, 19). The current knowledge on the characteristics of COPD is that in addition to airway obstruction, muscle, and weight loss are also prevalent (20). Therefore, levels of ALT may reflect the overall health status that contributes to the progression of COPD.

AST has a strong correlation with ALT and is generally known to be less specific to the liver than ALT because of its wide distribution. This study showed a U-shaped association of AST with COPD. However, few prior studies have reported associations between AST levels and COPD risk. Several meta-analyses demonstrated that levels of AST were significantly higher in patients with obstructive sleep apnoea and severe coronavirus disease 2019 (COVID-19) than in the general population (21, 22). The results from an animal study showed that liver steatosis promoted liver mitochondrial biogenesis in response to hypoxia (23). In addition, we found that the associations of ALT and AST levels with COPD risk were observed in never-smokers. A large cross-sectional study reported that smoking intensity was inversely associated with AST and ALT activities in individuals not drinking alcohol (24). The mechanism linking ALT, AST, and smoking is unclear. One hypothesis for this association is that hepatocellular vulnerability and the level of injury differ according to smoking status (24).

Regarding GGT, our finding of a positive association between circulating GGT levels and the risk of COPD is consistent with previous studies (25). Some case-control studies also indicated that serum GGT levels increased in individuals with stable COPD and were significantly higher in individuals with exacerbated COPD (26, 27). One possible explanation is that oxidative stress and inflammation have been demonstrated to be important amplifying mechanisms in COPD, and serum GGT is a sensitive biomarker of oxidative stress (28). Unexpectedly, we observed that the association of GGT with COPD risk was stronger among previous smokers in the stratified analysis. Nicotine might induce the elevation of GGT, and inflammation has a major role in the increase in GGT linked with smoking (24). Furthermore, airway inflammation causes structural changes that increase with COPD severity and last even after smoking cessation (1).

ALP, a hydrolytic enzyme mainly distributed in the liver and bone, is used as an indicator for skeletal monitoring in clinical practice (29). Skeletal muscle dysfunction is a severe complication in patients with COPD. It exacerbates COPD by decreasing the function of the ventilatory muscle groups (30). Muscle integrity reflects overall nutritional status and is thus indirectly linked to COPD. In our analysis, higher ALP levels were related to an increased risk of COPD, and the associations were stronger in young individuals, male individuals, frequent alcohol drinkers, and overweight individuals. The sex interaction might be related to oestrogen, which can effectively inhibit osteoblast apoptosis and bone resorption (31). A European-based population study also supported our findings that alcohol intake was inversely associated with ALP levels, although no significant effect was observed for extremely low alcohol intake (32). Sustained alcohol intake could lead to mild biliary obstruction, thereby reducing the number of ALP-producing cells (32). Additionally, although ageing and low BMI have been acknowledged as independent risk factors contributing to COPD mortality (1), the relationship between overweight and COPD risk remains controversial. The COPDGene study reported that obesity was prevalent in COPD patients and was associated with worse COPD-related outcomes (33). One possible hypothesis for this finding is that weight gain may not be exactly representative of increased muscle mass (30).

The inverse relationship of TBIL with COPD risk was fairly consistent with several prospective studies (8, 34). However, a bidirectional Mendelian randomization analysis showed no causal relation between TBIL and COPD (35). Although higher TBIL levels within the normal range have been demonstrated to decrease the risk of cardiovascular disease through antioxidant and anti-inflammatory effects (36), the underlying mechanism for the protective effects of TBIL in COPD progression is unclear. The results from animal models indicated that the effects of TBIL inhibiting lipid peroxidation might improve the lung function of COPD patients (37). Oxidative stress may further influence chronic lung inflammation. Furthermore, the UK primary care study showed that significantly obese individuals had relatively lower TBIL levels (8). A cross-sectional study based on the Japanese population reported that TBIL levels decreased only in women with BMI ≥ 27.5 kg/m^2^ (38). These studies supported our findings on the interaction of TBIL with BMI for COPD risk.

Although there have been no previous studies on the association between ALB and COPD progression, a meta-analysis including 6 studies demonstrated that severe COVID-19 patients had a higher prevalence of COPD and were significantly associated with decreased ALB levels (21). In clinical practice, circulating ALB is commonly measured to assess protein nutritional adequacy. Some studies suggested that higher ALB levels had a protective effect on the myocardium and were independently associated with muscle strength (39, 40). ALB levels were higher in males than females among young adults and declined with age (41). Liver injury caused by alcohol consumption commonly results in a decreased ability to synthesize protein (32). Furthermore, ALB is a major component of TP. Our analysis suggested that the inverse associations of ALB and TP with COPD risk were fairly consistent, which may indicate that the associations were mainly attributed to the role of ALB.

Previous studies have demonstrated that the liver-lung axis plays a key role in regulating lung inflammation (6). Our data also indicated a link between multiple liver function markers and COPD. A better understanding of the liver-lung axis may provide valuable insights for clinical intervention in COPD patients. The typical feature of COPD progression is a progressive loss in health status and deteriorating symptoms, with acute exacerbations linked to a higher mortality risk (1). Although exacerbations are generally shorter in duration, they also have a negative impact on the organism. COPD patients with recurrent exacerbations are often in poorer health status, and patients hospitalized for COPD exacerbations also have a poor long-term prognosis. Considering the complexity of COPD comorbidity and prognosis, managing acute exacerbation of COPD is a significant event in clinical care.

The major strengths of our study include its prospective design and long-term follow-up. The large sample size allowed us to minimize residual confounding by comprehensively adjusting COPD risk factors. In addition, all biomarkers were measured using standardized methods in the same laboratory with strict third-party IQC, minimizing potential measurement error. Several limitations of this study should also be acknowledged. First, our analysis used a single measure of circulating liver function markers that may not reflect the levels during the entire follow-up. However, the relatively high ICCs calculated from repeated measurements of blood samples provide reproducibility of liver function markers. Second, as an observational study, the possibility of reverse causality cannot be ruled out. However, the sensitivity analysis showed that the results were stable after excluding baseline hepatitis disease or diabetes or the first two years of follow-up. Third, to minimize selection bias, approximately a quarter of the UK Biobank participants were excluded, including 10% for prevalent COPD at baseline and 15% for missing liver function marker data or outliers in measurement. Finally, participants in the UK Biobank are predominantly European Caucasians, which limits the generalizability of our findings to other ethnicities.

In this large prospective population-based study, all seven circulating liver function markers were associated with the risk of COPD. These findings support the importance of liver function in COPD and may spur future studies to elucidate the role of the liver-lung axis in COPD development.

## Data availability statement

The data that support the findings of this study are available from the UK Biobank, but restrictions apply to the availability of these data, which were used under license for the current study and are not publicly available. Data are, however, available from the authors upon reasonable request and with the permission of the UK Biobank. Requests to access the datasets should be directed to https://www.ukbiobank.ac.uk/, via access@ukbiobank.ac.uk.

## Ethics statement

The UK Biobank study was approved by the North West Multi-centre Research Ethics Committee (06/MRE08/65). The patients/participants provided their written informed consent to participate in this study.

## Author contributions

XF and XW have full access to all the data in the study and take responsibility for the integrity of the data and the accuracy of the data analysis. Conception and design of the study: JZ and XF. Statistical analysis: HG and XW. Drafting of the manuscript: WD and HG. Critical revision of the manuscript for important intellectual content: ZZ, HY, PL, LC, JS, YL, DH, RT, and MW. All authors contributed to the article and approved the submitted version.
