# Supplementary material for: Circulating liver function markers and the risk of COPD in the UK Biobank
Source: Front Endocrinol (Lausanne). 2023 Mar 22;14:1121900. doi: 10.3389/fendo.2023.1121900 (PMC10073719; doi:10.3389/fendo.2023.1121900)
Supplement: Supplementary file 1 [file DataSheet_1.docx]

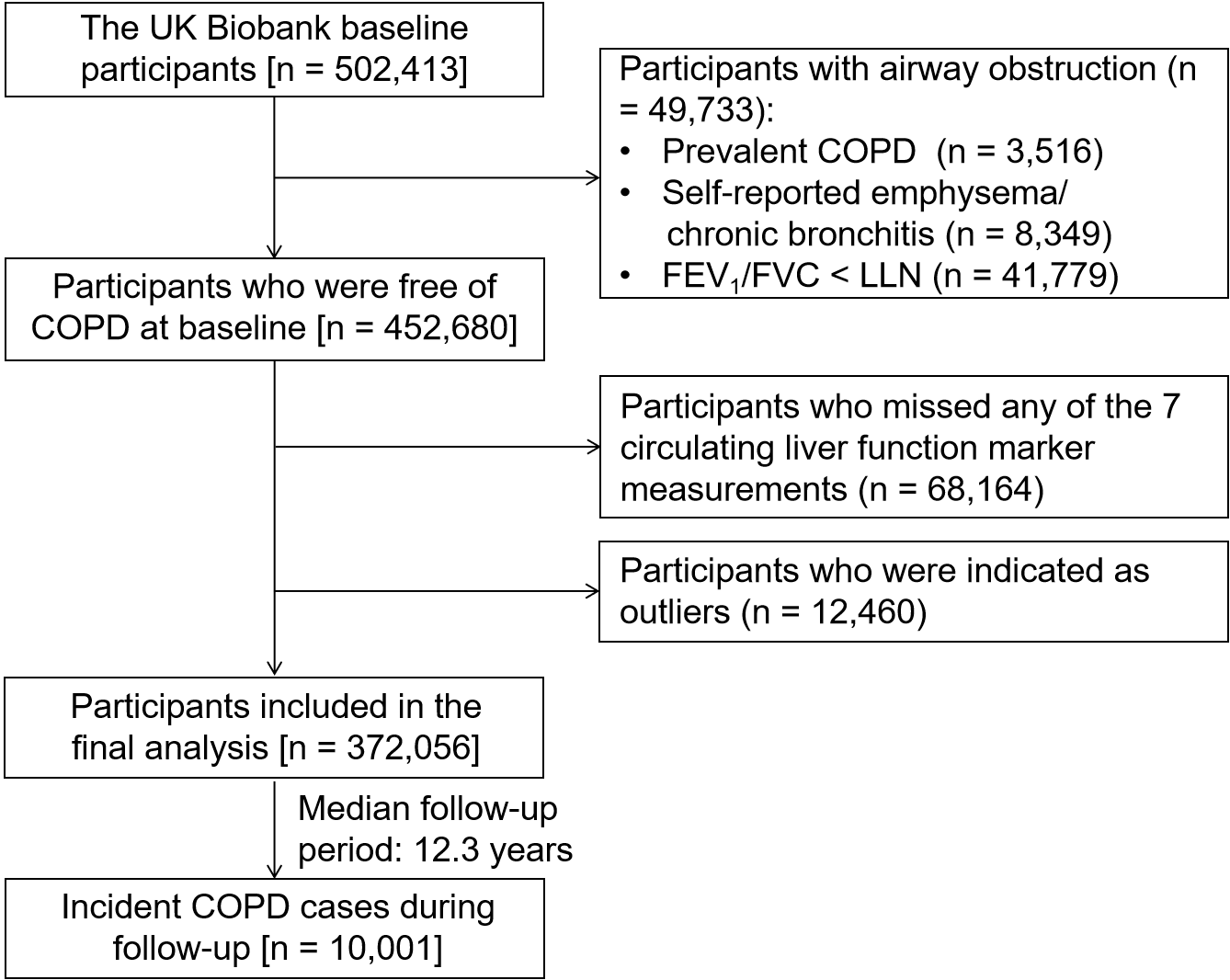


**Supplementary Figure 1. Flow chart of participant selection in the UK Biobank**

Abbreviations: COPD, chronic obstructive pulmonary disease; FEV_1_, forced expiratory volume in the first second; FVC, forced vital capacity; LLN, lower limit of normal.

| **Supplementary Table 1. Normal range for circulating liver function markers and the percentage of this study participants within the normal range^a^** | | |
| --- | --- | --- |
| **Biomarkers** | **Normal range** | **Percentage of participants within the normal range** |
| **Alanine aminotransferase (ALT)** | 7-52 U/L | 97.14% |
| **Aspartate aminotransferase (AST)** | 13-39 U/L | 95.67% |
| **Gamma-glutamyl transferase (GGT)** | 9-64 U/L | 90.77% |
| **Alkaline phosphatase (ALP)** | 34-104 U/L | 84.58% |
| **Total bilirubin (TBIL)** | 5-17 µmol/L | 88.33% |
| **Albumin (ALB)** | 35-57 g/L | 99.95% |
| **Total protein (TP)** | 64-89 g/L | 98.71% |
| ^a^Normal range is based on Beckman Coulter AU5800 determined reference range for circulating liver function markers (https://www.beckmancoulter.com/support/tech-docs) | | |

| **Supplementary Table 2. Age-adjusted Spearman correlation coefficients^a^** | | | | | | | | | | |
| --- | --- | --- | --- | --- | --- | --- | --- | --- | --- | --- |
|  | **ALT** | **AST** | **GGT** | **ALP** | **TBIL** | **ALB** | **TP** | **Age** | **BMI** | **CRP** |
| **ALT** | 1 | 0.69 | 0.58 | 0.16 | 0.12 | 0.15 | 0.13 | 0.03 | 0.33 | 0.15 |
| **AST** |  | 1 | 0.40 | 0.12 | 0.15 | 0.17 | 0.19 | 0.12 | 0.11 | 0.04 |
| **GGT** |  |  | 1 | 0.19 | 0.09 | 0.13 | 0.16 | 0.10 | 0.33 | 0.26 |
| **ALP** |  |  |  | 1 | -0.12 | -0.005 | 0.12 | 0.18 | 0.16 | 0.27 |
| **TBIL** |  |  |  |  | 1 | 0.21 | 0.08 | 0.01 | -0.07 | -0.18 |
| **ALB** |  |  |  |  |  | 1 | 0.47 | -0.13 | -0.12 | -0.19 |
| **TP** |  |  |  |  |  |  | 1 | -0.07 | 0.03 | 0.09 |
| **Age** |  |  |  |  |  |  |  | 1 | 0.07 | 0.12 |
| **BMI** |  |  |  |  |  |  |  |  | 1 | 0.44 |
| **CRP** |  |  |  |  |  |  |  |  |  | 1 |
| Abbreviations: ALT, alanine aminotransferase; AST, aspartate aminotransferase; GGT, gamma-glutamyl transferase; ALP, alkaline phosphatase; TBIL, total bilirubin; ALB, albumin; TP, total Protein; BMI, body mass index; CRP, C-reactive protein  ^a^All correlation coefficients are age adjusted except for those with age itself and all *P* values < 0.05. | | | | | | | | | | |

| **Supplementary Table 3. Intraclass correlation coefficients of circulating liver function markers measured in repeated samples^a^** | |
| --- | --- |
|  | **Intraclass correlation coefficient (95% CI)** |
| **Alanine aminotransferase (ALT)** | 0.50 (0.49, 0.51) |
| **Aspartate aminotransferase (AST)** | 0.49 (0.47, 0.50) |
| **Gamma-glutamyl transferase (GGT)** | 0.61 (0.59, 0.62) |
| **Alkaline phosphatase (ALP)** | 0.72 (0.71, 0.73) |
| **Total bilirubin (TBIL)** | 0.73 (0.73, 0.74) |
| **Albumin (ALB)** | 0.46 (0.44, 0.47) |
| **Total protein (TP)** | 0.46 (0.44, 0.47) |
| ^a^Intraclass correlation coefficients were calculated in the subsample of participants with repeat measurements of circulating liver function markers (n = 11892) | |

| **Supplementary Table 4. Sensitivity analyses of the association between circulating levels of liver function markers and COPD risk in the UK Biobank^a^** | | | | | | | | |
| --- | --- | --- | --- | --- | --- | --- | --- | --- |
|  | **No. of COPD cases** | **ALT** | **AST** | **GGT** | **ALP** | **TBIL** | **ALB** | **TP** |
| **The primary findings for the total cohort** | |  |  |  |  |  |  |  |
| HR (95% CI), decile 10 vs. 1 | 10001 | 0.92 (0.84-1.01) | 1.04 (0.95-1.13) | 1.45 (1.31-1.62) | 1.31 (1.19-1.45) | 0.82 (0.75-0.89) | 0.74 (0.67-0.81) | 0.96 (0.88-1.04) |
| HR (95% CI), per 1-SD increment | 10001 | 0.98 (0.96-1.01) | 1.03 (1.01-1.05) | 1.10 (1.08-1.12) | 1.09 (1.07-1.11) | 0.95 (0.93-0.98) | 0.89 (0.87-0.91) | 0.99 (0.97-1.01) |
| **Excluding the first two years of follow-up (n=735)** | | |  |  |  |  |  |  |
| HR (95% CI), decile 10 vs. 1 | 9266 | 0.93 (0.84-1.03) | 1.05 (0.96-1.15) | 1.41 (1.26-1.58) | 1.30 (1.17-1.44) | 0.82 (0.74-0.90) | 0.75 (0.69-0.83) | 0.97 (0.89-1.06) |
| HR (95% CI), per 1-SD increment | 9266 | 0.98 (0.96-1.01) | 1.03 (1.01-1.05) | 1.10 (1.08-1.12) | 1.09 (1.07-1.11) | 0.96 (0.93-0.98) | 0.90 (0.88-0.92) | 0.99 (0.97-1.01) |
| **Excluding participants with abnormally low or high levels of circulating liver function markers^b^** | | | | |  |  |  |  |
| HR (95% CI), decile 10 vs. 1 | - | 0.90 (0.82-0.96) | 0.98 (0.89-1.09) | 1.34 (1.12-1.61) | 1.25 (1.11-1.40) | 0.84 (0.73-0.96) | 0.74 (0.68-0.81) | 0.98 (0.90-1.07) |
| HR (95% CI), per 1-SD increment | - | 0.96 (0.94-0.99) | 1.01 (0.98-1.03) | 1.15 (1.10-1.19) | 1.13 (1.09-1.17) | 0.97 (0.93-0.99) | 0.89 (0.88-0.91) | 1.00 (0.98-1.02) |
| **Excluding participants with hepatitis and other liver/hepatobiliary disease at recruitment (n=8680)** | | | | |  |  |  |  |
| HR (95% CI), decile 10 vs. 1 | 9529 | 0.92 (0.84-1.02) | 1.03 (0.94-1.13) | 1.43 (1.28-1.60) | 1.33 (1.20-1.48) | 0.81 (0.74-0.89) | 0.72 (0.66-0.79) | 0.95 (0.87-1.04) |
| HR (95% CI), per 1-SD increment | 9529 | 0.98 (0.96-1.01) | 1.03 (1.01-1.05) | 1.10 (1.08-1.12) | 1.09 (1.07-1.12) | 0.95 (0.93-0.98) | 0.89 (0.87-0.91) | 0.99 (0.97-1.01) |
| **Excluding participants with diabetes at recruitment (n=7413)** | | |  |  |  |  |  |  |
| HR (95% CI), decile 10 vs. 1 | 9366 | 0.93 (0.84-1.02) | 1.06 (0.97-1.16) | 1.47 (1.32-1.64) | 1.36 (1.22-1.51) | 0.83 (0.75-0.91) | 0.73 (0.66-0.80) | 0.99 (0.91-1.08) |
| HR (95% CI), per 1-SD increment | 9366 | 0.99 (0.96-1.01) | 1.04 (1.02-1.06) | 1.11 (1.09-1.13) | 1.10 (1.07-1.12) | 0.96 (0.93-0.98) | 0.89 (0.87-0.91) | 0.99 (0.98-1.02) |
| Excluding participants with asthma at recruitment (n=37573) | | | | | |  |  |  |
| HR (95% CI), decile 10 vs 1 | 7355 | 0.91 (0.81-1.01) | 1.04 (0.94-1.15) | 1.48 (1.30-1.68) | 0.81 (0.73-0.90) | 1.35 (1.20-1.51) | 1.01 (0.91-1.11) | 0.74 (0.66-0.82) |
| HR (95% CI), per 1-SD increment | 7355 | 0.97 (0.95-0.99) | 1.03 (1.01-1.06) | 1.11 (1.09-1.13) | 0.96 (0.93-0.98) | 1.02 (1.01-1.03) | 1.01 (0.99-1.03) | 0.83 (0.80-0.87) |
| Excluding participants with atherosclerotic disease at recruitment (n=8199) | | | |  |  |  |  |  |
| HR (95% CI), decile 10 vs 1 | 9252 | 0.93 (0.84-1.02) | 1.03 (0.94-1.12) | 1.44 (1.29-1.61) | 0.84 (0.76-0.92) | 1.28 (1.16-1.42) | 0.96 (0.88-1.05) | 0.75 (0.68-0.83) |
| HR (95% CI), per 1-SD increment | 9252 | 0.99 (0.97-1.01) | 1.03 (1.01-1.05) | 1.10 (1.08-1.12) | 0.96 (0.94-0.98) | 1.01 (1.01-1.02) | 0.99 (0.97-1.01) | 0.84 (0.81-0.87) |
| Adjustment for additional cholesterol lowering medication for the total cohort | | | |  |  |  |  |  |
| HR (95% CI), decile 10 vs 1 | 10001 | 0.88 (0.80-0.96) | 1.00 (0.92-1.09) | 1.39 (1.25-1.55) | 0.80 (0.73-0.88) | 1.33 (1.21-1.47) | 0.95 (0.87-1.03) | 0.70 (0.64-0.76) |
| HR (95% CI), per 1-SD increment | 10001 | 0.97 (0.95-0.99) | 1.02 (1.00-1.04) | 1.09 (1.07-1.11) | 0.95 (0.93-0.97) | 1.02 (1.01-1.02) | 0.99 (0.97-1.01) | 0.81 (0.79-0.84) |
| **Adjustment for additional dietary and nutritional supplement intake^c^** | | | |  |  |  |  |  |
| HR (95% CI), decile 10 vs. 1 | 10001 | 0.92 (0.83-1.01) | 1.05 (0.96-1.15) | 1.44 (1.29-1.60) | 1.31 (1.18-1.45) | 0.82 (0.75-0.90) | 0.74 (0.68-0.81) | 0.96 (0.88-1.04) |
| HR (95% CI), per 1-SD increment | 10001 | 0.98 (0.96-1.00) | 1.03 (1.01-1.05) | 1.10 (1.08-1.12) | 1.09 (1.07-1.11) | 0.96 (0.94-0.98) | 0.89 (0.88-0.91) | 0.99 (0.97-1.01) |
| Abbreviations: ALT, alanine aminotransferase; AST, aspartate aminotransferase; GGT, gamma-glutamyl transferase; ALP, alkaline phosphatase; TBIL, total bilirubin; ALB, albumin; TP, total Protein; BMI, body mass index; CRP, C-reactive protein  ^a^Multivariable Cox regression model with age as the underlying time scale was used and adjusted for sex, race (white, nonwhite, unknown), fasting status (<8 hours, ≥8 hours), assessment center and age at recruitment (continuous), Townsend deprivation index (continuous), education level (college/university degree, noncollege/university degree, unknown), body mass index (continuous), total physical activity (continuous), alcohol consumption frequency (never or special occasions only, once a month to twice a week, three times a week to daily), smoking status (never, previous, current-pack-years <10, current-pack-years ≥10 and <20, current-pack-years ≥20 and <30, current-pack-years ≥30), C-reactive protein (continuous), family history of respiratory disease (no, yes, unknown), passive smoking (never, <20 hours a week, ≥20 hours a week), PM_2.5_ (continuous), occupations at risk of COPD (no, yes, unknown).  ^b^The NO. of COPD cases for excluding participants with abnormally low or high levels of circulating liver function markers were 9710 for ALT, 9432 for AST, 8570 for GGT, 8674 for TBIL, 7570 for ALP, 9784 for TP, and 9987 for ALB.  ^c^Additional adjustment for raw vegetable intake (continuous), cooked vegetable intake (continuous), fresh fruit intake (continuous), dried fruit intake (continuous), frequency of poultry consumption and livestock consumption (never, less than once a week to once a week, twice a week to daily), coffee intake (continuous), vitamin supplements (any of vitamin A, vitamin B, vitamin C, vitamin D, vitamin E, folate, and multivitamins, or none), and mineral and other dietary supplements (any of calcium, zinc, iron, selenium, glucosamine, and fish oil, or none). | | | | | | | | |
